# Pneumatic Displacement and Anti-VEGF Therapy for Submacular Hemorrhage in Neovascular Age-Related Macular Degeneration: A Retrospective Study

**DOI:** 10.3390/jcm14093154

**Published:** 2025-05-02

**Authors:** Hikaru Ota, Jun Takeuchi, Ryo Nonogaki, Kazuma Tamura, Taro Kominami

**Affiliations:** 1 Department of Ophthalmology, Nagoya University Graduate School of Medicine, 65 Tsurumai, Showa, Nagoya 466-8550, Aichi, Japan; 2 Department of Ophthalmology, Kyorin University School of Medicine, 6-20-2 Shinkawa, Mitaka 181-8611, Tokyo, Japan

**Keywords:** neovascular age-related macular degeneration, anti-VEGF, pneumatic displacement, submacular hemorrhage

## Abstract

**Background/Objectives:** Submacular hemorrhage (SMH) associated with neovascular age-related macular degeneration (nAMD) can lead to significant vision loss, and the optimal management strategy remains uncertain. This study aimed to evaluate the efficacy and safety of pneumatic displacement (PD) without tissue plasminogen activator (t-PA) for SMH secondary to nAMD. **Methods:** A retrospective analysis was conducted on 22 eyes with SMH secondary to nAMD treated with PD without t-PA. Best-corrected visual acuity (BCVA), central retinal thickness (CRT), number of intravitreal injections, and postoperative complications were assessed at baseline and follow-up. Multiple logistic regression analyses were used to identify factors associated with visual outcomes. **Results:** In the 22 eyes that completed the 6-month follow-up, BCVA (logMAR) was 0.88 ± 0.46 at baseline and 0.76 ± 0.63 at 6 months (*p* = 0.24). In the 15 eyes with 12-month follow-up, BCVA improved significantly from 0.92 ± 0.47 at baseline to 0.56 ± 0.51 at 12 months (*p* = 0.01). CRT significantly decreased at 3 months (*p* < 0.01). During this period, patients received an average of 8.13 ± 2.90 intravitreal anti-vascular endothelial growth factor (VEGF) injections. A shorter duration from symptom onset to treatment was associated with better visual outcomes (*p* = 0.02). Postoperative vitreous hemorrhage occurred in 31.8% of cases. **Conclusions:** PD without t-PA, in combination with anti-VEGF therapy, improved visual outcomes over 12 months. Early intervention and continuous anti-VEGF administration appear to be key factors in optimizing treatment outcomes. Further studies are needed to establish standardized treatment protocols for SMH associated with nAMD.

## 1. Introduction

Submacular hemorrhage (SMH) is a serious vision-threatening complication of neovascular age-related macular degeneration (nAMD), a leading cause of blindness in older adults [1,2]. SMH occurs when blood accumulates in the subretinal space, disrupting retinal metabolism and leading to irreversible damage through iron toxicity, mechanical stress on photoreceptors, and the formation of fibrotic barriers that impair retinal function [3]. The incidence of SMH with vision loss in patients undergoing anti-vascular endothelial growth factor (anti-VEGF) therapy is estimated to be 0.46% per year, according to a 10-year observational study [4].

Various treatment approaches have been proposed to manage SMH and reduce its impact on visual outcomes, including anti-VEGF monotherapy, pneumatic displacement (PD) with or without tissue plasminogen activator (t-PA), and vitrectomy [5,6,7,8,9]. However, there is no consensus on the optimal treatment strategy, particularly for large SMH secondary to nAMD. PD without t-PA, combined with intravitreal anti-VEGF therapy, has shown promising results in smaller studies, but its efficacy and safety remain underexplored, particularly in larger hemorrhages [7].

To address this gap, this study evaluates the efficacy and safety of PD without t-PA for the treatment of SMH secondary to nAMD. Furthermore, we investigate prognostic factors influencing visual outcomes and identify potential predictors of complications, providing new insights into optimizing treatment strategies for patients with SMH.

## 2. Materials and Methods

### 2.1. Study Design

The study was approved by the Ethics Committee of Nagoya University Hospital (approval number: 2021-039923469) and conducted in accordance with the tenets of the Declaration of Helsinki. The medical records of patients diagnosed with SMH secondary to nAMD at Nagoya University Hospital between January 2014 and June 2023 were retrospectively reviewed.

### 2.2. Subjects

This study included a consecutive series of eyes with large and thick SMH, defined as lesions exceeding 2 disk diameters (DD) in size and a retinal detachment greater than 100 μm at the fovea, secondary to nAMD. These eyes were treated with PD without t-PA within 14 days of symptom onset. The exclusion criteria were an underlying retinal condition other than nAMD, such as retinal macroaneurysm, symptom onset more than 14 days before treatment, a follow-up period of less than 6 months, or a history of vitrectomy before the initial presentation. All patients underwent comprehensive ophthalmic examinations to support a multimodal imaging-based diagnosis. These evaluations included best-corrected visual acuity (BCVA) measurements using the logarithm of the minimum angle of resolution (logMAR), fundus photography, fluorescein angiography, and indocyanine green angiography (Heidelberg Engineering, Heidelberg, Germany). Spectral-domain optical coherence tomography (SD-OCT) was also conducted using the same system. Central retinal thickness (CRT) was manually measured on B-scan OCT images using a caliper tool integrated into the OCT system and was defined as the distance from the inner limiting membrane to Bruch’s membrane. Data were obtained at 3, 6, and 12 months.

### 2.3. Treatment Methods

After paracentesis of 0.2–0.3 mL, 0.3 mL of pure sulfur hexafluoride (SF6) was injected intravitreally through the pars plana. Patients were instructed to maintain a prone position for 3 days. Aflibercept (2 mg, 0.05 mL) or ranibizumab (0.5 mg, 0.05 mL) was injected intravitreally via the pars plana under sterile conditions within 1 week before or after the gas injection. The selection between aflibercept and ranibizumab was at the physician’s discretion. These procedures were performed using 30- or 34-gauge needles.

Subsequent treatment consisted of regular follow-up visits with continued intravitreal injections of either ranibizumab or aflibercept, administered at the physician’s discretion based on the presence of hemorrhage, signs of active choroidal neovascularization on multimodal imaging, such as subretinal or intraretinal fluid, or both, as assessed at each examination.

### 2.4. Statical Analysis

All statistical analyses were performed using SPSS software (version 28.0.0.0; IBM Corp., Armonk, NY, USA). Decimal BCVA values were converted to logarithmic logMAR units for analysis. The Shapiro–Wilk test was used to assess the normality of data distribution. The Mann–Whitney U test, Fisher’s exact test, and Wilcoxon matched-pairs signed-rank test were used for statistical comparisons. Bonferroni correction was applied to adjust for multiple testing when comparing baseline and follow-up data, following previous reports [10]. The corrected *p*-value thresholds were calculated by dividing 0.05 by the number of independent tests. For BCVA and CRT analyses across multiple time points, results were considered statistically significant if they fell below the Bonferroni correction threshold: *p* < 0.025 (i.e., 0.05/2) for the 6-month follow-up and *p* < 0.016 (i.e., 0.05/3) for the 12-month follow-up. Multiple logistic regression analysis was also performed. Data are presented as the mean ± standard deviation, and *p*-values for statistical tests, except for those subject to Bonferroni correction, were considered statistically significant at *p* < 0.05.

## 3. Results

### 3.1. Clinical Characteristics of Patients

A total of 22 eyes from 22 patients who underwent PD for nAMD and had a minimum follow-up of 6 months were included in the study. The nAMD subtypes comprised polypoidal choroidal vasculopathy (PCV) in 17 eyes (77.2%) and type 1 macular neovascularization (MNV) in 5 eyes (22.7%). At baseline, the mean BCVA was 0.88 ± 0.46 logMAR, the mean SMH diameter was 5.84 ± 3.48 DD, and the mean SMH height was 482.2 ± 245.1 μm. Among the 22 eyes, 15 patients continued follow-up for at least 12 months. The baseline characteristics of patients at the 6- and 12-month follow-ups are presented in Table 1.

### 3.2. Treatment Outcomes

**Figure 1:**
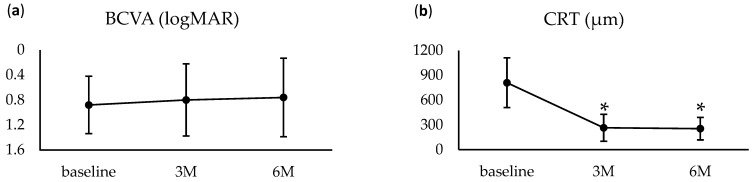
Changes in the clinical findings for cases with ≥6 months of follow-up (22 eyes). (**a**) Changes in visual acuity from baseline to 6 months. Although a trend toward improvement was observed, the change was not statistically significant. (**b**) Changes in central retinal thickness (CRT) from baseline to 6 months. CRT significantly decreased from month 3 and remained reduced through month 6. * Significance threshold: *p* < 0.025 vs. baseline, Wilcoxon signed-rank test with Bonferroni correction.

Among the 22 eyes that completed 6 months of follow-up, BCVA improved from 0.88 ± 0.46 at baseline to 0.80 ± 0.58 at 3 months (*p* = 0.40) and 0.76 ± 0.63 at 6 months (*p* = 0.24), though these changes were not statistically significant (Figure 1a). Improvement in visual acuity was observed in 15 eyes (68.2%) over 6 months. CRT significantly decreased from 810.2 ± 300.8 μm at baseline to 265.7 ± 163.2 at 3 months (*p* < 0.01) and 255.0 ± 135.5 μm (*p* < 0.01) at 6 months (Figure 1). During this period, patients received an average of 5.32 ± 1.81 intravitreal anti-VEGF injections, with aflibercept used in 20 eyes and ranibizumab in 2 eyes.

### 3.3. One-Year Outcomes

**Figure 2:**
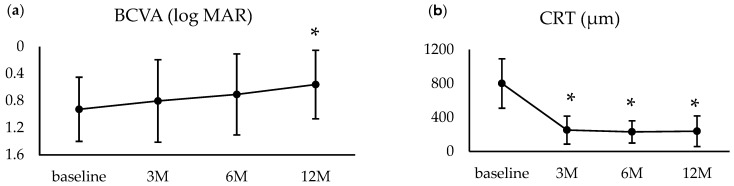
Changes in clinical findings for cases with ≥12 months of follow-up (15 eyes). (**a**) Changes in visual acuity from baseline to 12 months. While changes were not statistically significant up to 6 months, significant improvement was observed at 12 months. (**b**) Changes in central retinal thickness (CRT) from baseline to 12 months. CRT significantly decreased from month 3 and remained reduced through month 12. * Significance threshold: *p* < 0.016 vs. baseline, Wilcoxon signed-rank test with Bonferroni correction.

In the 15 eyes that completed 12 months of follow-up, BCVA improved from 0.92 ± 0.47 at baseline to 0.80 ± 0.61 at 3 months (*p* = 0.27) and 0.71 ± 0.60 at 6 months (*p* = 0.08), though these improvements were not statistically significant. However, by 12 months, BCVA showed a significant improvement to 0.56 ± 0.51 (*p* = 0.01). CRT significantly decreased from 801.6 ± 290.9 μm at baseline to 252.9 ± 165.1 μm at 3 months (*p* < 0.01) and remained stable thereafter (Figure 2). Over the 12-month follow-up, the mean number of intravitreal anti-VEGF injections administered was 8.13 ± 2.90, with aflibercept used in 13 eyes and ranibizumab in 2 eyes.

### 3.4. Complications

Seven eyes (31.8%) developed vitreous hemorrhage (VH), of which two (9.1%) also experienced rhegmatogenous retinal detachment (RRD). The mean interval between the initial treatment and VH onset was 21.8 ± 10.1 days (range: 1–33 days). The intervals from initial treatment to RRD development were 27 and 28 days, respectively. Among the seven eyes with VH, five cases were mild and resolved spontaneously. The remaining two cases, which developed RRD accompanied by visually significant VH, were treated with pars plana vitrectomy. In one of these cases, the RRD involved the macula and was associated with a macular hole. Despite undergoing vitrectomy, the visual outcome was poor (BCVA: 0.52 at baseline, 1.52 at 6 months). In the other case, the RRD was caused by a small superior temporal retinal break with macular involvement. The retina was successfully reattached following vitrectomy, resulting in a favorable visual outcome (BCVA: 0.10 at baseline, 0 at 6 months). Additionally, one eye (4.55%) experienced a recurrence of SMH 33 days post-treatment and underwent additional treatment with an SF6 injection.

### 3.5. Factors Associated with Visual Outcomes

To assess the clinical factors influencing visual outcomes 6 months after the initial treatment, we compared the baseline characteristics between eyes that showed visual improvement (n = 15) and those that did not (n = 7). Univariate analysis demonstrated that eyes with improvement had a significantly shorter duration from symptom onset to treatment compared to those without improvement (5.11 ± 3.86 vs. 9.38 ± 5.29 days, *p* = 0.01). Multivariate logistic regression analysis, adjusted for time to treatment, baseline BCVA, SMH diameter, and SMH height, confirmed that a shorter duration until treatment was a significant predictor of better visual prognosis (*p* = 0.02) (Table 2). OCT findings revealed that ellipsoid zone (EZ) disruption was present in all 22 eyes at baseline. At 6 months, EZ disruption remained in 19 eyes and was absent in 3 eyes. Eyes with persistent EZ disruption at 6 months had significantly worse visual acuity than those without (0.92 ± 0.58 vs. 0.02 ± 0.05, *p* < 0.01).

### 3.6. Factors Associated with VH

To identify clinical factors associated with the incidence of VH following PD, we compared the baseline characteristics of eyes that developed VH (n = 7) and those that did not (n = 15). Univariate analysis showed that eyes with VH had a significantly larger SMH diameter compared to those without VH (DD: 8.61 ± 4.77 vs. 4.55 ± 1.68, *p* = 0.02). However, multivariate logistic regression analysis, adjusted for treatment duration, baseline BCVA, SMH diameter, and SMH height, did not identify any significant predictors of VH incidence (Table 3).

## 4. Discussion

This retrospective study evaluated the treatment outcomes of PD without t-PA in combination with continued intravitreal anti-VEGF injections for SMH associated with nAMD. Our findings demonstrated a gradual improvement in visual acuity, which became statistically significant at 12 months. Typically, in nAMD without large SMH, continuous anti-VEGF therapy leads to maximal visual improvement within 3 months, after which the primary goal shifts to maintaining visual acuity [11]. However, our findings suggest that in cases of large SMH associated with nAMD, continued anti-VEGF therapy following PD may lead to a slower but more sustained improvement in visual acuity over time. This observation is important for patient counseling, as physicians should inform patients that visual recovery may extend beyond 3 months, potentially improving adherence to long-term treatment.

To date, few studies have examined PD without t-PA in combination with anti-VEGF therapy for SMH. Wakabayashi et al. reported gradual visual acuity improvement from 3 months to 1 year post-treatment [7]. Our study similarly observed improvement at 3 months, but statistical significance was not reached until 1 year. In contrast, Wakabayashi et al. reported a marked improvement in visual acuity as early as 3 months after treatment. PCV has been associated with a better visual prognosis than other SMH subtypes secondary to nAMD [12]. Furthermore, PCV has been shown to achieve better visual and disease control with anti-VEGF therapy than other subtypes [13,14]. Wakabayashi et al. focused exclusively on PCV, whereas 22.7% of our study population were patients with type 1 MNV, which may have contributed to the differences in treatment outcomes.

Patients who exhibited visual improvement had a significantly shorter duration from symptom onset to treatment than those who did not. Furthermore, multivariate analysis identified the time from onset to treatment as a significant independent predictor of visual prognosis. Several reports have indicated that a shorter duration between symptom onset and treatment is associated with better visual outcomes, and our study yielded similar results [15,16]. Basic research has suggested that retinal damage from SMH occurs through multiple mechanisms: in the short term, the clot disrupts the outer retinal layers, while in the long term, iron toxicity and metabolic impairment of the retinal pigment epithelium contribute to retinal degeneration. Widespread irreversible damage to the outer retina has been reported to occur within 7–14 days [4,15]. The findings of our study align with this pathophysiological framework and suggest that early therapeutic intervention is critical for patients with SMH associated with nAMD. Other factors, such as the maximum diameter and height of the SMH, as well as the nAMD subtype, have been previously reported as prognostic indicators for visual outcomes [15,16,17,18]. However, in our study, none of these factors were significant predictors of visual prognosis.

In this study, seven eyes (31.8%) developed VH, and two of these eyes (9.1%) also experienced RRD. The incidence of VH after PD has been reported to range from 11.4% to 25.0%, and the frequency observed in our study was higher than previously reported [7,16,19,20,21,22]. To investigate potential factors associated with postoperative VH, we analyzed all cases, including mild VH that did not affect visual function, which may have contributed to the higher observed VH incidence. Additionally, our findings showed that eyes that developed postoperative VH had a significantly larger preoperative SMH diameter than those that did not, a relationship observed in previous studies [17]. Several mechanisms have been proposed to explain VH development following SMH, including retinal necrosis caused by extensive hemorrhage, which allows red blood cells to permeate, and the formation of small retinal tears due to subretinal or intraretinal hemorrhage, leading to blood migration into the vitreous cavity [21,22]. In the present study, one case of retinal detachment associated with a macular hole was observed, suggesting that the development of a macular hole may represent an additional mechanism by which subretinal hemorrhage can enter the vitreous cavity. These mechanisms are more likely to occur in cases of extensive hemorrhage with a large volume of subretinal blood. However, multivariate analysis did not identify a significant association between SMH diameter and VH occurrence, highlighting the need for further investigation in a larger cohort.

Various treatment strategies for SMH have been reported, including anti-VEGF monotherapy, PD with or without t-PA, and vitrectomy. However, there is currently no established consensus on the optimal treatment approach. Shin et al. compared intravitreal aflibercept monotherapy with PD combined therapy and found that in SMH cases ≥450 μm in thickness, the group receiving PD had a better visual prognosis [6]. Similarly, Inoue et al. recommended PD or vitrectomy for SMH cases ≥2 DD in size [12]. Several studies have also reported the effectiveness of combining t-PA with PD [23,24,25]. However, conflicting findings exist. Cakir et al. compared PD alone with PD combined with t-PA and found no significant difference in visual prognosis between the two groups [26]. Similarly, Fujikawa et al. reported no significant differences in visual outcomes or SMH displacement between PD alone and PD with t-PA [27]. Given these inconsistencies, further prospective studies with larger sample sizes are required to determine whether combining PD with t-PA provides additional benefits. Several studies have reported improved visual outcomes after vitrectomy [28,29]. However, basic research suggests that vitrectomy increases vitreous clearance, potentially reducing the long-term efficacy of intravitreal anti-VEGF injections in nAMD management [30]. Moreover, a randomized controlled trial comparing PD and vitrectomy did not find a clear superiority of vitrectomy over PD [31]. In contrast, Jeong et al. reported that vitrectomy resulted in a better visual prognosis than PD in patients with extensive SMH [32]. Although no definitive consensus exists regarding the optimal treatment, our findings suggest that PD without t-PA remains a reasonable option for SMH, offering visual improvement with minimal invasiveness. However, patients with a longer delay from symptom onset to treatment tended to have poorer visual outcomes. In such cases, further investigation is warranted to assess the potential benefits of combining PD with t-PA or considering vitrectomy as an alternative treatment strategy.

This study has several limitations. First, it was a retrospective study with a small sample size. The small sample size was primarily due to the rarity of SMH associated with nAMD and the fact that some patients experienced delays between symptom onset and treatment, making them ineligible for PD. As a result of these limitations, the evaluation of visual prognosis, incidence of complications, and potential prognostic factors may have been affected. Second, visual function was assessed solely using BCVA, without evaluating other functional aspects such as metamorphopsia or localized visual field defects. As a result, postoperative visual impairment may have been underestimated. Finally, this was a single-arm study, which precluded direct comparisons between treatment strategies and limited the ability to determine the optimal management approach for SMH associated with nAMD.

## 5. Conclusions

This study demonstrated that PD followed by continuous anti-VEGF therapy was associated with improved visual outcomes in patients with SMH secondary to nAMD. Additionally, we found that a longer duration from symptom onset to treatment was associated with poorer visual outcomes, and that eyes developing VH after treatment had a larger extent of SMH at the time of treatment initiation. These findings highlight the importance of early PD intervention and continuous anti-VEGF administration in achieving better outcomes. Currently, there is no established consensus on the optimal treatment strategy for SMH associated with nAMD. Further studies with larger sample sizes and extended follow-up periods are needed to refine treatment guidelines. This study provides valuable data on the treatment outcomes of PD without t-PA, a strategy that has been rarely reported, and may contribute to future clinical decision-making.

## Figures and Tables

**Table 1:** Baseline characteristics of patients followed up for 6 and 12 months.

	6 Months’ Follow-Up	12 Months’ Follow-Up
Number of eyes/ patients	22/22	15/15
Age (year; mean ± SD, range)	75.5 ± 9.25	74.0 ± 9.88
Sex, eyes (%)		
Male	19 (86.4)	13 (15.4)
Female	3 (13.6)	2 (13.3)
Subtype, eyes (%)		
Type 1 MNV	5 (22.7)	2 (13.3)
PCV	17 (77.3)	13 (86.7)
Anticoagulant treatment, yes (%)	2 (9.10)	1 (6.67)
Duration of SMH (days; mean ± SD, range)	7.63 ± 5.12	8.40 ± 5.43
Previous anti-VEGF treatment, yes (%)	8 (36.4)	5 (33.3)
BCVA (logMAR; mean ± SD)	0.88 ± 0.46	0.92 ± 0.47
CRT (μm; mean ± SD, range)	810.3 ± 300.8	801.6 ± 290.9
Size of SMH (DD; mean ± SD, range)	5.84 ± 3.48	5.58 ± 3.59
Height of retinal detachment(μm; mean ± SD, range)	482.2 ± 245.1	473.1 ± 286.6

BCVA, best-corrected visual acuity; DD, disk diameter; logMAR, the logarithm of the minimum angle of resolution; SMH, submacular hemorrhage; VEGF, vascular endothelial growth factor; and SD, standard deviation.

**Table 2:** Univariate and multivariate analysis comparing baseline factors between eyes with visual improvement (n = 15) and those without improvement (n = 7) at 6 months.

	Patients with Visual Improvement (n = 15)	Patients Without Visual Improvement (n = 7)	*p* (Univariate)	*p* (Multivariate)	Odds Ratio (95% Cl)
Age (year; mean)	77.7 ± 7.70	70.7 ± 11.1	0.10		
Sex			0.23		
Male	14 (63.6)	5 (22.7)			
Female	1 (6.67)	2 (9.1)			
Subtype, eyes (%)			0.48		
Type 1 MNV	4 (18.1)	1 (4.50)			
PCV	11 (50.0)	6 (27.3)			
Duration of SMH (days)	5.11 ± 3.86	9.38 ± 5.29	<0.01	0.02	0.51–0.95
BCVA (logMAR)	0.89 ± 0.37	0.85 ± 0.64	0.86	0.65	0.14–23.2
Size of SMH (DD)	5.60 ± 2.53	6.33 ± 5.19	0.90	0.40	0.79–1.82
Height of retinal detachment (μm)	420.4 ± 246.8	545.7 ± 287.4	0.42	0.29	0.99–1.00

MNV, macular neovascularization; PCV, polypoidal choroidal neovasculopathy; SMH, submacular hemorrhage; DD, disk diameter; and BCVA, best-corrected visual acuity.

**Table 3:** Univariate and multivariate analyses comparing baseline factors between eyes with VH (n = 7) and those without VH (n = 15) after treatment.

	Patients with VH (n = 7)	Patients Without VH (n = 15)	*p* (Univariate)	*p* (Multivariate)	Odds Ratio (95% Cl)
Age (year; mean)	74.7 ± 9.59	75.8 ± 9.41	0.81		
Sex			0.71		
Male	6 (27.3)	13 (59.1)			
Female	1 (4.55)	2 (9.1)			
Subtype, eyes (%)			0.11		
Type 1 MNV	0 (0)	5 (22.7)			
PCV	7 (31.8)	10 (45.5)			
Duration of SMH (days)	8.00 ± 6.40	7.47 ± 4.66	0.83	0.22	0.42–1.22
BCVA (logMAR)	0.84 ± 0.56	0.90 ± 0.42	0.80	0.26	0.01–3.93
Size of SMH (DD)	8.61 ± 4.77	4.55 ± 1.68	0.02	0.06	0.95–6.02
Height of retinal detachment (μm)	409.0 ± 343.3	484.2 ± 221.9	0.95	0.42	1.00–1.01

MNV, macular neovascularization; PCV, polypoidal choroidal neovasculopathy; SMH, submacular hemorrhage; DD, disk diameter; and BCVA, best-corrected visual acuity.

## Data Availability

The datasets used in this study are available from the corresponding author upon request.
